# Microbiome-Derived Metabolites in Allogeneic Hematopoietic Stem Cell Transplantation

**DOI:** 10.3390/ijms22031197

**Published:** 2021-01-26

**Authors:** Riccardo Masetti, Daniele Zama, Davide Leardini, Edoardo Muratore, Silvia Turroni, Patrizia Brigidi, Andrea Pession

**Affiliations:** 1 Pediatric Oncology and Hematology “Lalla Seràgnoli”, Pediatric Unit, IRCCS Azienda Ospedaliero-Universitaria di Bologna, 40138 Bologna, Italy; riccardo.masetti5@unibo.it (R.M.); daniele.zama@aosp.bo.it (D.Z.); edoardo.muratore@studio.unibo.it (E.M.); andrea.pession@unibo.it (A.P.); 2 Unit of Microbial Ecology of Health, Department of Pharmacy and Biotechnology, University of Bologna, 40126 Bologna, Italy; silvia.turroni@unibo.it; 3 Department of Medical and Surgical Sciences (DIMEC), University of Bologna, 40126 Bologna, Italy; patrizia.brigidi@unibo.it

**Keywords:** gut microbiome, hematopoietic stem cell transplantation, metabolome, graft-vs-host disease

## Abstract

The gut microbiome has emerged as a major character in the context of hematopoietic stem cell transplantation. The biology underpinning this relationship is still to be defined. Recently, mounting evidence has suggested a role for microbiome-derived metabolites in mediating crosstalk between intestinal microbial communities and the host. Some of these metabolites, such as fiber-derived short-chain fatty acids or amino acid-derived compounds, were found to have a role also in the transplant setting. New interesting data have been published on this topic, posing a new intriguing perspective on comprehension and treatment. This review provides an updated comprehensive overview of the available evidence in the field of gut microbiome-derived metabolites and hematopoietic stem cell transplantation.

## 1. Introduction

Allogeneic hematopoietic stem cell transplantation (allo-HSCT) is a well-established treatment for a variety of hematologic malignancies, immune disorders and metabolic diseases [1]. Allo-HSCT often represents the only possible curative therapy, however it is hampered by high morbidity and mortality rates for an array of complications, including bloodstream infection and graft-versus-host disease (GvHD) [2]. Recently, the gut microbiome (GM) has emerged as a major contributor to the genesis of these complications and to transplant outcomes [3,4,5]. While this relationship has been extensively studied in terms of clinical correlations, the underlying biological processes still remain poorly understood [6]. A growing body of evidence is now focused on the role of metabolomics in the immune response regulation and in other host biochemical processes [7]. Interestingly, among the factors that modify fecal, plasmatic and urinary metabolites, the GM, alongside with diet, have emerged as the major determinants [8]. Analogously, metabolic activities of GM are affected by environmental factors and host activities. The latter include a complex crosstalk taking place in the intestinal mucosa, with the secretion of mucus, secretory IgA, antibacterial peptides and microRNA [9]. Hence it has been suggested that microbiome-derived metabolites could provide some insights in the complex relationship between the GM, immune system and intestinal microenvironment, particularly in the HSCT setting [10]. To address this issue, we conducted a narrative literature review of studies addressing the role of gut microbiota derived metabolites in allo-HSCT. Electronic databases, including PubMed, Google Scholar and EMBASE, were searched to identify relevant studies published up to December 2020. The search was restricted to English-language studies involving both humans, mice and pre-clinical models. Papers were selected independently by two authors independently, and a third author supervised the selection. Herein, we provide a comprehensive overview on the current knowledge of gut microbiome-derived metabolites and their role in determining relevant biological processes in HSCT (Table 1 and Table 2).

## 2. Fiber-Derived Metabolites—Short-Chain Fatty Acids

**Figure 1:**
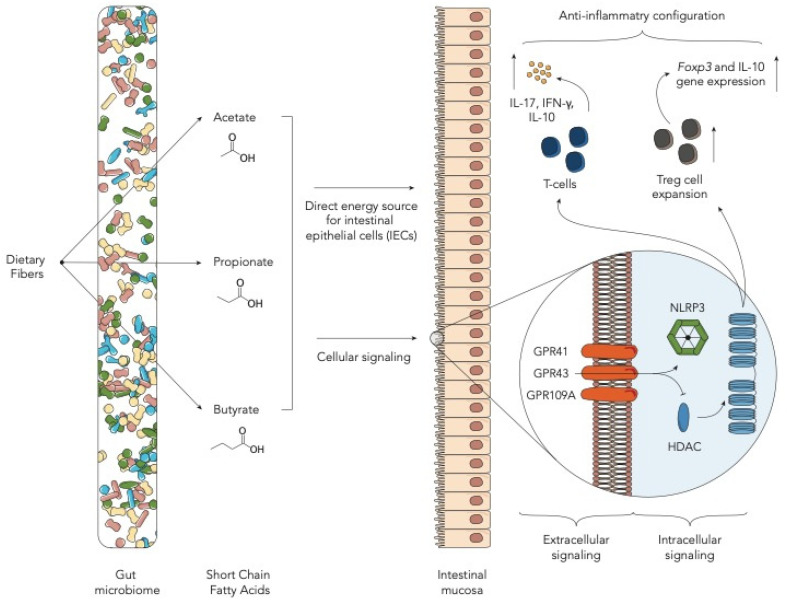
Schematic representation of the role of short chain fatty acids in the allogeneic hematopoietic stem cell transplantation (allo-HSCT) setting (HDAC—histone deacetylases; IEC—intestinal epithelial cells).

Short-chain fatty acids (SCFAs) are well-known bacterial products derived from the GM [32]. SCFAs, in particular acetate, propionate and butyrate, are the major products of the fermentative activity of GM in the cecum and colon on dietary fibers [33]. This conversion involves most of the enteric bacteria through the establishment of syntrophic consortia that operate several specific metabolic reactions [33]. Butyrate, an important SCFA in the allo-HSCT context, derives from two main routes, namely, phosphotransbutyrylase/butyrate kinase and butyryl-CoA:acetate CoA-transferase. The former is carried out mainly by *Coprococcus eutactus* and *Coprococcus comes*, the latter by *Eubacterium rectale*, *Roseburia* spp., *Eubacterium hallii*, *Anaerostipes* spp., *Coprococcus catus* and *Faecalibacterium prausnitzii* [34,35]. SCFAs have shown to have important immune-modulatory functions acting as both intracellular and extracellular signaling molecules targeting different receptors such as GPR43, GPR41 and GPR109A [10,36]. Intracellularly, these molecules, specifically butyrate, serve as direct inhibitors of histone deacetylases (HDACs), enzymes needed to convert chromatin from a permissive to a repressive structure [37]. Through HDAC inhibition, SCFAs regulate T-cells by directly promoting their differentiation into T cells producing IL-17, IFN-γ and IL-10. Besides, they also promote de novo differentiation and expansion of regulatory T cells [38,39]. Among this cell subset, butyrate, by enhancing histone H3 acetylation, induces the upregulation of Foxp3 locus and IL-10 gene expression [38,40]. Butyrate has also been demonstrated to have a direct effect on the intestinal mucosa, serving as an energy source for intestinal epithelial cells (IECs) [41] (Figure 1). Focusing on the allo-HSCT setting, the ability of SCFAs, specifically butyrate, to inhibit HDACs has been associated with a reduction in acute GvHD (aGvHD) [42]. Data on mouse models proved that butyrate improves IEC integrity, decreases apoptosis and mitigates aGVHD. Mathewson et al. have demonstrated that butyrate levels decrease in the mouse intestine after HSCT, resulting in a significant reduction in the histone H4 acetylation degree and in functional impairment of IECs [11]. Furthermore, they demonstrated that higher levels of butyrate were associated with reduced severity of aGvHD and that they could be achieved with the administration of specific clostridial strains [11]. Similar results were found by Stein-Thoeringer et al. in a cohort of 1325 allo-HSCT patients and in a pre-clinical mouse model. They observed that post-transplant loss of Clostridia was accompanied by a significant reduction in fecal butyrate in patients and mice with GvHD [12]. Fujiwara et al. further confirmed the importance of SCFAs in aGVHD protection in a mouse model and highlighted the role of the SCFA-specific receptor GPR43 and the signaling pathway involving NLRP3 inflammasome activation in IECs [13]. In particular, they found that administration of two main SCFAs, butyrate and propionate, in wild-type mice, reduced aGvHD. Interestingly, administration of butyrate and propionate in GPR43^−/−^ mice produced lower and no effect in reducing aGvHD, respectively. No effect was seen for acetate, the most present SCFA in our gut [13]. These data together underscore the important role of GPR43 in modulating the beneficial effect of SCFAs and that butyrate has both a GPR43-dependent and independent effect. Later studies on human HSCT patients found overlapping results. Payen et al. analyzed the SCFA content in 35 adult HSCT patients who went on to develop aGvHD compared to the same number of non-aGvHD controls. They found that acetate, propionate and butyrate, as well as the total SCFA level were lower in patients with stage 2–3 aGvHD [18]. Interestingly, a significant difference only for butyrate was found with controls even in patients with grade 1 aGvHD, further confirming the very pivotal role of this metabolite [11]. These data were confirmed by Romick-Rosendale et al. in an exclusive pediatric population. They prospectively analyzed fecal samples from 42 pediatric patients undergoing HSCT, showing a reduction in butyrate, propionate and acetate in patients who went on to develop aGvHD [19]. Besides, the authors found some differences compared to the mouse model. Particularly, they found a progressive decline in SCFAs in the first 14 days after HSCT, with a significant reduction in butyrate and propionate. Moreover, by analyzing the RNA expression levels of several butyrate transporters, they found that, in aGvHD patients, some of them are decreased, as previously described (e.g., GPR41-FFAR3), but many others are increased (e.g., GPR41-FFAR2) or not significantly changed. Lastly, the authors suggested a role of another SCFA, formate, as a possible marker for the Enterobacteriaceae family within the Proteobacteria phylum [19]. The alteration in SCFA levels can also be ascribed to the HSCT-related dysbiosis [43]. Romick-Rosendale et al., in their aforementioned study, also pointed out the impact of antibiotics on SCFAs, showing that a greater number of days of antibiotic was associated with lower levels of butyrate and propionate [19]. A recent study by Markey et al. suggested a role of SCFAs also in the context of chronic GvHD (cGvHD). They analyzed both fecal and plasma SCFA concentrations from nine and ten cGvHD patients and non-matched controls, respectively. Metabolic alterations were found to be present both in plasma and in fecal samples of patients with cGvHD. Specifically, plasma concentrations of butyrate, propionate, hexanoate and isobutyrate were significantly lower in patients who went on to develop cGvHD compared with controls [20]. Taxonomic analysis revealed that the presence of Lachnoclostridium, Clostridium and, to a lesser degree, Faecalibacterium was associated with reduced incidence of cGvHD [20]. Galloway-Peña et al. analyzed the butyrate content in longitudinal fecal specimens from 44 HSCT patients. While in this cohort butyrate was not statistically associated with GvHD, it was found that patients experiencing bloodstream infections within 30 days after HSCT had a significantly lower level of butyrate, further suggesting the role of this SCFA in maintaining endothelial integrity [21]. Regarding viral infections, Haak et al. analyzed a cohort of 360 adult patients undergoing HSCT in a single institution, focusing on viral lower respiratory tract infections following allo-HSCT. They showed that patients with higher abundance of butyrate-producing bacteria were five-fold less likely to develop such infections. The same relationship was found with the fecal concentration of butyrate, propionate, acetate and desaminotyrosine [22]. All these data together underscore the crucial role of SCFAs in gut homeostasis and in the onset of HSCT complications, and, besides, raise the possibility that host diets may play a role. Yoshifuji et al. analyzed the impact of resistant starch and a commercially available prebiotic mixture, GFO, given from the start of the conditioning regimen until day +28 after HSCT on gut microbiota products. Interestingly, oral supplementation resulted in reduced incidence of all aGvHD grades, a higher prevalence of butyrate-producing bacteria at day +28 and a consequent maintained or increased fecal butyrate concentration [23]. Similar results were obtained by D’Amico et al. in our pediatric cohort. We prospectively analyzed the impact of enteral nutrition (EN) compared to parenteral nutrition (PN) on microbiological outcomes. We found that EN patients were significantly enriched in butyrate, acetate and propionate compared to subjects who received PN. Moreover, well-known health-associated genera capable of producing SCFAs were restored in the EN group during the post-HSCT recovery, namely Faecalibacterium, Dorea, Blautia, Bacteroides, Parabacteroides and Oscillospira [24].

## 3. Amino Acid-Derived Metabolites

### 3.1. Tryptophan-Derived AhR Ligands

**Figure 2:**
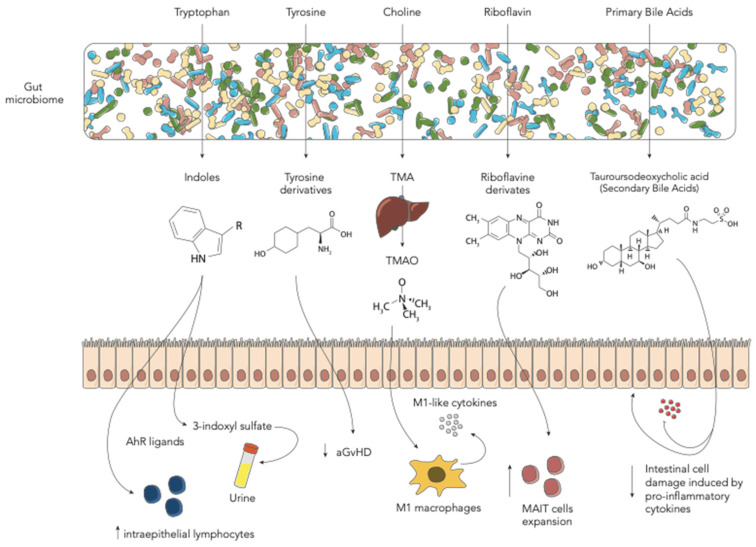
Schematic representation of the main gut microbiome-derived metabolites in the allo-HSCT setting (aGvHD—acute graft-vs-host disease; MAIT—mucosal-associated invariant T cells; TMA—trimethylamine; TMAO—trimethylamine N-oxide).

Other important GM metabolites are tryptophan and its derivatives (Figure 2). Tryptophan is an essential amino acid, which our organism cannot synthesize, and therefore must be supplied with food [44]. Degradation of tryptophan can be ascribed to many intestinal bacteria, such as Fusobacterium, Bacteroides and *Enterococcus faecalis*, which have the ability to convert tryptophan into indole and its derivatives. Other reviews have addressed this issue [44]. Mounting evidence has shown that GM-produced tryptophan catabolites, such as indole and its derivatives, are important signaling molecules in host-microbial crosstalk. These metabolites can act on different human physiological processes, such as gut mucosal homeostasis and reactivity, gastrointestinal motility, insulin secretion, anti-oxidative and anti-inflammatory processes. Each function seems to be regulated by different receptors, namely pregnane X receptor [45], G protein-coupled receptors [46], IL-10 receptors [47], Nrf2 receptors [48], PPAR [49] and aryl hydrocarbon receptor (AhR). The latter is a ligand-dependent transcription factor known to strongly interact with our immune system [50,51]. It has been demonstrated that AhR deficiency or the lack of AhR ligands reduced intraepithelial lymphocyte numbers and compromised the control of the GM in the intestinal lumen [52]. Another study showed that the tryptophan catabolite indole-3-aldehyde, via the AhR pathway, provides colonization resistance to the yeast Candida albicans and reduces intestinal inflammation [53]. Less is known about these metabolites in the HSCT context. Swimm et al. analyzed the levels of indoles and its derivatives during HSCT in mouse models. They found that mice exposed to lethal radiation as well as to chemotherapeutic conditioning regimens had lower urinary levels of 3-indoxyl sulfate (3-IS) [14]. This metabolite is derived from the tryptophan-derived indole, metabolized by the liver and excreted into the urine, known to be a potent endogenous agonist for the human aryl hydrocarbon receptor [54,55]. Mice colonized with an *Escherichia coli* strain unable to degrade tryptophan had lower 3-IS levels and lower post-transplant overall survival. The authors then tested the possibility of modulating the level of metabolites with the supplementation of indole-3-carboxaldehyde, an indole derivative. Very interestingly, they found that this treatment reduced gut epithelial damage and GvHD-related mortality by decreasing inflammatory cytokines, suggesting an important immunomodulatory role of such GM degradation products [14]. In particular, the protective effect exerted by indoles seems to be mediated by T-helper 17 responses in the intestinal tract and by IL-22-mediated effects on stem cells [53]. Furthermore, transcriptomic data on mice indicate that indoles activate IFN1 responses only in the context of GvHD, suggesting a link with concomitant immune-mediated inflammation [14]. Similar results were found by Weber et al. in human patients. In a cohort of 131 adult patients receiving allo-HSCT, they tested urinary levels of 3-IS within the first 28 days after transplant and found that low 3-IS levels were associated with higher transplant-related mortality and worse outcomes, mainly due to gastrointestinal (GI) GvHD. Interestingly, authors also demonstrated that 3-IS urinary levels could be correlated with GM diversity and with a higher presence of E. rectale and Ruminococcaceae, taxa belonging to the Clostridia class [25]. In this study, urinary levels of 3-IS thus appeared to be a possible marker for assessing the presence of a healthy GM configuration. DeFilipp et al. also highlighted the possibility of modulating GM-related metabolites. They performed fecal microbiota transplantation (FMT) by a third-part donor in 13 adult patients receiving allo-HSCT. In these patients they observed an increase of Clostridiales abundance and a significant increase in 3-IS urinary concentrations, further confirming previous data in both mouse and human patients [26]. Michonneau et al. analyzed metabolic alterations in two independent monocentric and multicentric cohorts composed of 43 and 56 patients, respectively, receiving allogeneic-HSCT from an HLA-identical sibling donor. Focusing on metabolic changes at GvHD onset, they observed that the main contributors were bile acids, plasmalogens, tryptophan and arginine metabolites. Among these, indolepropionate, a GM-derived compound from tryptophan was the only metabolite less frequently detected in the multicentric cohort [27]. In both cohorts it was found that the tryptophan-derived 3-IS was involved in the GvHD-related metabolic alterations [27]. These results, alongside with the one found by Swimm et al. and Weber et al., seem to disagree with other evidence in patients with end stage renal disease. In fact, 3-IS is also a uremic toxin known to be associated with adverse outcomes in patients with renal failure. In this particular clinical setting, monocytes, responding to 3-IS through the AhR pathway, produce increased levels of TNF-α. The resulting pro-inflammatory environment and immune dysfunction seems related to vascular endothelial cell damage and to the pathogenesis of cardiovascular disease [56]. Probably, the different clinical setting and the presence of allo-immune mediate inflammation may play an important role in modulating the host response to 3-IS. This disagreement should be addressed in future research.

### 3.2. Tyrosine-Derived Metabolites

Tyrosine is a non-essential amino acid found in food, well known as it is involved in the synthesis of catecholamines [57]. The GM in the large intestine is involved in tyrosine fermentation resulting in the production of many derived metabolites [58]. Their role in many brain physiological and pathological conditions has been widely described [59]. Li et al. focused on the role of this amino acid in a mouse model receiving HSCT. They analyzed the metabolic profile of mice receiving bone marrow only and bone marrow with donor T cells as a model for in-vivo aGvHD. Strikingly, the analysis indicated that the low level of tyrosine in the gut was likely to be correlated with the occurrence and development of aGvHD and that tyrosine-derived metabolites were inversely correlated with the presence of Blautia and Enterococcus [15]. Furthermore, the authors found that dietary supplementation with tyrosine was able to ameliorate aGvHD in the early stages, restore GM diversity and modify the relative abundance of specific taxa [15]. Reikvam et al. analyzed the pre-transplant metabolic profile in 86 adult patients receiving allo-HSCT [28]. In order to figure out whether a certain metabolic pattern could predict the onset of aGvHD, they extensively studied a total of 766 metabolites in the serum of patients with and without aGvHD. Among the large number of produced results, they found that tyrosine metabolism was altered in patients with aGvHD. They also hypothesize that the observed differences in tyrosine as well as tryptophan, lysine and phenylalanine suggest a pivotal role of the pre-HSCT GM metabolic configuration in predicting the development of aGvHD [28].

### 3.3. Choline-Derived Metabolites

Wu et al. recently reported the role of choline-derived trimethylamine N-oxide (TMAO) in the HSCT context [16]. This metabolite is already well known to play an important role in vascular inflammation and endothelial dysfunction, contributing to the genesis of atherosclerosis and thrombosis [60]. TMAO derives from the oxidation by hepatic flavin monooxygenases of trimethylamine (TMA), which is a GM metabolite of betaine, L-carnitine, choline and other choline-containing compounds, which are present in the diet [61]. The main involved bacterial strains are *Anaerococcus hydrogenalis*, *Clostridium asparagiforme*, *Clostridium hathewayi*, *Clostridium sporogenes*, *Edwardsiella tarda*, *Escherichia fergusonii*, *Proteus penneri* and *Providencia rettgeri* [62]. Both TMAO and choline were found to be associated in mouse model with an enhanced allogeneic GvHD reaction [16]. Furthermore, the authors demonstrate that TMAO induces the expression of M1 macrophages and M1-like cytokines both in tissues and in bone marrow in an NLRP3-dependent fashion [16,63].

## 4. Riboflavin (Vitamin B2)-Derived Metabolites

Another family of GM-derived metabolites found to have an important role in the HSCT context are riboflavin-based precursors. These metabolites are produced by a wide range of bacteria including *E. coli*, Staphylococcus aureus and *Pseudomonas aeruginosa*, and have a positive effect in expanding a particular T-cell subtype known as mucosal-associated invariant T cells (MAIT) [64,65]. In particular, MAIT cells respond to vitamin B2/B9–derived metabolites presented by MR1, an MHC class I–like molecule, producing IFN-g, IL-17, releasing cytotoxic granules and antibacterial products [66]. These cells are known to play a pivotal role in the GvHD genesis after HSCT, both in a mouse model and in humans [67,68,69,70]. However, there is no direct evidence that fecal riboflavin concentration has an impact on HSCT-related complications or outcome. Konuma et al. analyzed the GM expression of enzymes involved in the riboflavin synthesis pathway in 121 patients receiving unrelated cord blood transplant. Intriguingly, they found that within the KEGG pathway, the amounts the genes ribB and ribA, encoding two important involved enzymes, namely, 3,4-dihydroxy-2-butanone-4-phosphate synthase and GTP cyclohydrolase II, were higher in patients with MAIT reconstitution after HSCT [29].

## 5. Bile Acids

Bile acids (BAs) are cholesterol-derived amphipathic molecules participating in the digestion and absorption of fat in the diet. Primary BAs are synthesized in the liver, conjugated with glycine or taurine and secreted in the intestinal tract [71]. Secondary BAs result from bacterial transformation in the gut by removing glycine or taurine residues from primary BAs not reabsorbed by IECs [72,73]. BAs were demonstrated to be altered in a small cohort of HSCT patients developing gut aGvHD. This was related to intestinal malabsorption but no correlation was made with GM [74]. Haring et al. focused on BA metabolism in an HSCT mouse model. Firstly, they demonstrated that BAs were decreased, and BA receptor expression was altered by HSCT [17]. Among BAs they found that GM-derived tauroursodeoxycholic acid was capable of reducing intestinal cell damage induced by pro-inflammatory cytokines and improving HSCT outcomes in a prophylactic setting. Notably, while tauroursodeoxycholic acid was found to protect the intestinal epithelium by directly affecting intestinal cells, its administration did not lead to a change in microbial composition [17]. In humans, both Michonneau et al. and Reikvam et al. found alterations in BAs after HSCT, in particular the latter found a decrease of secondary BAs in patients developing aGvHD [27,28].

## 6. Polyamines and Breath Metabolites

**Figure 3:**
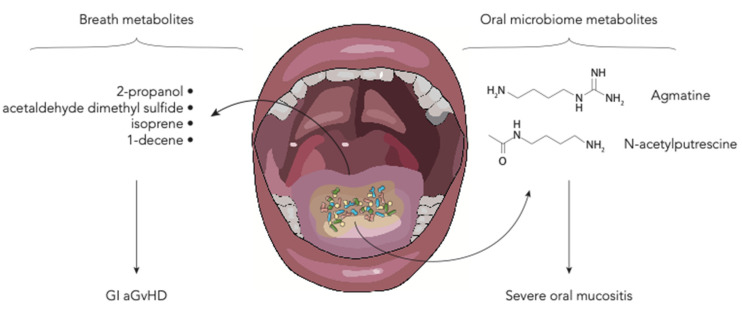
Schematic representation of the oral and breath metabolites in the allo-HSCT setting (aGvHD—acute graft-vs-host disease; GI—gastrointestinal).

The oral microbiome is known to play a major role in the genesis of several pathological conditions and mucositis in HSCT patients [75,76] (Figure 3). Shouval et al. demonstrated that also oral microbiome-derived metabolites are altered in patients developing oral mucositis during HSCT. They analyzed the salivary metabolic profile of patients with and without severe oral mucositis, showing a reduction in N-acetylputrescine and agmatine, metabolites involved in the polyamine pathway [30]. Polyamines are small polycationic molecules produced by commensal bacteria with a wide array of biological functions including preservation of mucosal barrier integrity [77]. New interesting insights were described by Hamilton et al. regarding breath metabolites. They analyzed volatile organic compounds in 19 HSCT adult patients. They found that in patients developing GI GvHD, levels of five compounds, namely 2-propanol, acetaldehyde, dimethyl sulfide, isoprene, and 1-decene, were altered [31]. Some evidence suggests that GM metabolism may be involved in the production of these compounds [78].

## 7. Conclusions

GM-derived metabolites have emerged as crucial players in mediating crosstalk between GM and host in allo-HSCT recipients. Several questions should still be addressed in the upcoming studies. Firstly, the different metabolic profiles should be more precisely characterized and the relationship between specific bacterial strains and derived metabolites should be investigated. These data should be accomplished with ‘–omics’ approaches, including metabolomics, metagenomics and metatranscriptomics. Alongside with the aforementioned metabolites, many others have been demonstrated to have a role in human homeostasis and should thus be investigated in the HSCT setting [10,79]. Future collaborative studies on larger cohorts will also clarify whether specific metabolic profiles could be associated with allo-HSCT outcomes as it has been demonstrated for GM diversity [3]. Lastly, the different metabolic patterns between children and adults should be directly addressed, considering the differences in GM configuration [80] and HSCT outcomes [81]. Certainly, these data pose a new intriguing field of research and substantial opportunities for the near future. GM-derived metabolites might serve as a feasible surrogate marker for microbiome characterization that may be clinically useful to predict HSCT-related risk [25]. Furthermore, the modulation of GM-derived metabolites should also appear as a target for therapeutic interventions. These should include diet, which is known to represent the main strategy to modulate microbial products [8], emphasizing the importance of nutritional support during HSCT [24,43,82,83]. Other strategies should also be tested in order to modulate metabolites, such as probiotics, prebiotics and other oral supplements alongside FMT. In conclusion, GM-derived metabolites have proven to be an important field of research in the HSCT setting, also appearing as a promising therapeutic target for the near future.

## Figures and Tables

**Table 1:** Summary of studies investigating the role of microbiome-derived metabolites in HSCT setting in mouse model.

Metabolites	Results	References
Fiber-Derived Metabolites—Short-Chain Fatty Acids		
Butyrate	Butyrate can improve IEC integrity, decrease apoptosis and mitigate GvHD. Administration of Clostridiales strain leads to higher butyrate levels.	[11]
Butyrate	Post-transplant enterococcal domination and loss of Clostridiales were associated with a reduction in butyrate in mice developing GvHD.	[12]
Butyrate, propionate	Butyrate and propionate improve GvHD in mouse model. This effect is dependent on the presence of SCFA receptor GRP43.	[13]
Amino Acid-Derived Metabolites		
Tryptophan-derived AhR ligand		
Indoles and derivatives	GM derivatives, such as indole, limit intestinal inflammation and damage associated with myeloablative chemotherapy or radiation exposure and acute GvHD. Treatment with indole-3-carboxaldehyde can protect from gut damage in HSCT recipients.	[14]
Tyrosine-derived metabolites		
Tyrosine	Mice with aGvHD present lower levels of tyrosine. Oral administration of tyrosine can ameliorate aGvHD and modify GM configuration.	[15]
Choline-derived metabolites		
TMAO	TMAO augments allo-reactive T-cell proliferation and Th1 subtype differentiation mediated by the polarized M1 macrophages. This results in higher severity of GvHD.	[16]
Bile Acids		
Tauroursodeoxycholic acid (TUDCA)	BAs were altered after HSCT. Administration of exogenous TUDCA protects intestinal epithelium by inflammatory cytokines. TUDCA did not influence GM composition.	[17]

**Table 2:** Summary of studies investigating the role of microbiome-derived metabolites in HSCT setting in human.

Metabolites	Study Design	Results	References
Fiber-Derived Metabolites—Short-Chain Fatty Acids			
Butyrate	1325 allo-HSCT adult patients	Post-transplant enterococcal domination and loss of Clostridiales were associated with a reduction in butyrate in patients developing GvHD.	[12]
Butyrate, propionate, acetate	35 allo-HSCT adult aGvHD patients	Butyrate, propionate and acetate levels were lower in patients experiencing GvHD 2–3 compared to the control. Butyrate was low even in patents with GvHD 1.	[18]
Butyrate, propionate, acetate, formate	42 allo-HSCT pediatric patients	Butyrate, propionate, acetate decrease within the first 14 days after HSCT and are lower in patients developing GvHD. Formate is a possible marker for the *Enterobacteriaceae* family. Expression of butyrate transporters in GvHD is altered. Greater number of days of antibiotic was associated with lower levels of butyrate and propionate.	[19]
Butyrate, propionate, hexanoate, isobutyrate	10 allo-HSCT adult cGvHD patients	Plasma concentration of SCFAs reflects fecal content. Patents developing cGvHD present lower plasma concentration of butyrate, propionate, hexanoate, isobutyrate.	[20]
Butyrate	44 allo-HSCT adult patients	Butyrate levels were correlated with Shannon index and were low in patients experiencing bloodstream infections within 30 days after HSCT.	[21]
Butyrate, propionate, acetate, desaminotyrosine	360 allo-HSCT adult patients	Butyrate-producing bacteria and fecal SCFAs were associated with a protection from viral lower respiratory tract infections	[22]
Butyrate	99 allo-HSCT adult patients	Oral supplementation with resistant starch and commercially available prebiotic mixture, GFO, resulted in higher post-HSCT butyrate-producing bacteria and a maintained or increased fecal butyrate concentration.	[23]
Butyrate, propionate, acetate	20 allo-HSCT pediatric patients	Enteral nutrition resulted in higher fecal concentration of butyrate, propionate and acetate.	[24]
Amino Acid-Derived Metabolites			
Tryptophan-derived AhR ligand			
3-IS	131 allo-HSCT adult patients	Lower 3-IS urinary levels are associated with higher transplant-related mortality and worse outcome. 3-IS urinary levels are correlated with GM diversity and with a higher presence of *Eubacterium rectale* and *Ruminococcaceae*.	[25]
3-IS	13 allo-HSCT adult patients receiving FMT	FMT results in higher 3-IS urinary levels.	[26]
Indoxyl sulfate	Two cohort of 43 and 56 allo-HSCT adult patients	Tryptophan-derived AhR ligand 3-indoxyl sulfate was involved in the GvHD-related metabolic alterations.	[27]
Tyrosine-derived metabolites			
Tyrosine	86 allo-HSCT adult patients	In patients who develop aGvHD tyrosine metabolism was found to be altered. Other microbiome-derived metabolites (tryptophan, lysine, phenylalanine and secondary bile acids) were altered.	[28]
Riboflavin (Vitamin B2)-Derived Metabolites			
Riboflavin	121 allo-HSCT adult patients receiving CBT	Patients with post-HSCT MAIT cells reconstitution had a GM with higher expression of genes involved in the riboflavin synthesis pathway.	[29]
Polyamines and Breath Metabolites			
N-acetylputrescine, agmatine	184 allo-HSCT adult patients	Salivary metabolic profile of HSCT patients with and without severe oral mucositis (grade 0–1 vs. 3–4) was found to be different. Metabolites such as urea, 5-aminovalerate, N-acetylputrescine and agmatine, also show differences between the pre-transplant and the time of mucositis onset.	[30]
2-propanol, acetaldehyde, dimethyl sulfide, isoprene, and 1-decene	19 allo-HSCT adult patients	Comparing patients with and without GI GvHD, the former show modification in the levels of volatile organic compounds, namely 2-propanol, acetaldehyde, dimethyl sulfide, isoprene, and 1-decene.	[31]

## Abbreviations

3-IS	3-indoxyl sulfate
aGvHD	Acute graft-versus-host disease
Allo-HSCT	Allogeneic hematopoietic stem cell transplantation
BA	Bile acids
cGvHD	Chronic GvHD
EN	Enteral nutrition
FMT	Fecal microbiota transplantation
GI	Gastrointestinal
GM	Gut microbiome
GvHD	Graft-versus-host disease
HDAC	Histone deacetylases
IEC	Intestinal epithelial cells
MAIT	Mucosal-associated invariant T cells
PN	Parenteral nutrition
SCFA	Short-chain fatty acids
TMA	Trimethylamine
TMAO	Trimethylamine N-oxide
